# Synthesis and characterization of glyoxal-crosslinked chitosan with N-amino anthracene succinimide polymers for antimicrobial applications

**DOI:** 10.1186/s13065-025-01505-2

**Published:** 2025-05-22

**Authors:** Ahmed G. Taha, A. M. Hezma

**Affiliations:** 1 Department of Chemistry, Faculty of Science, South Valley University, Qena, 83523 Egypt; 2 Spectroscopy Department, Physics Research Institute, National Research Centre, Cairo, 12622 Egypt

**Keywords:** Chitosan, Glyoxal, N-amino-9,10-dihydro-anthracene-9,10-α,β-succinamid, Polymers, XRD, IR, Antibacterial applications

## Abstract

This paper presents the synthesis and characterization of a novel chitosan-derivative polymers by using glyoxal as acrosslinker between chitosan and N-amino-9,10-dihydro-anthracene-9,10-α,β-succinamide (AS) under our conditions. The generated polymers were characterized using X-ray diffraction (XRD) and infrared spectroscopy (IR). The results indicate the successful formation of new polymer structures with unique properties. The antimicrobial activity of Chs-Gly-AS and its modified derivatives was evaluated using the cup diffusion agar method, showing enhanced inhibition against bacteria and fungi. The results demonstrate promising antimicrobial efficacy, suggesting the potential use of these polymers in antimicrobial applications.

## Introduction

Biodegradable polymers referred to as herbal substances are presently utilized in a variety of industries, like tissue culture, biomedicine, farming, food, and clever fabrics. Biopolymers may be acquired from animal, plant, or herbal reassets and are taken into consideration a brand new technology of herbal substances [1–3]. Biopolymer substances have attracted interest because of their richness, sustainability, environmental friendliness, and biodegradability properties [4, 5]. It is likewise really well worth noting that herbal polymers may be acquired in good sized portions from diverse renewable reassets, while artificial polymers are derived from non-renewable petroleum resources [6]. Chitin is a herbal polymeric polysaccharide extracted from marine crustaceans, and chitosan is acquired through eliminating a part of the acetyl institution (typically greater than 60%) in chitin`s structure [7]. Chitosan (Chs) is used in lots of fields because of its biodegradability, biocompatibility, nontoxicity, non-adhesiveness, and film-forming capabilities [8, 9]. Chitosan has antibacterial, antimicrobial and antifungal activities, which can be of its different proper attributes [10]. The functional structure of chitosan includes six primary hydroxyl (OH) groups, three secondary hydroxyl (OH) groups, two amine (NH₂) groups, and a few acetyl groups along with glycosidic bonds. The − NH₂ and − OH groups within the chitosan molecule are highly reactive, allowing the synthesis of a wide range of chitosan derivatives through reactions with various chemical groups, which serve as key sites for chemical modifications [11–14].

Chitosan has crucial intrinsic residences like mucoadhesion, permeation enhancer, and antimicrobial residences [15]. –OH and -NH_2_ of chs are crucial for its residences like mucoadhesion, permeation enhancement, managed drug release, in situ gelation, and antimicrobial [16]. The insolubility of chitosan in water and maximum natural solvents has restrained its use in many fields [17]. To overcome these limitations, chemical modifications are employed to produce chitosan derivatives. These modifications improve the physicochemical properties of chitosan and broaden its potential applications [18].

Chitosan has been widely utilized in antibacterial research due to its remarkable antibacterial properties, excellent biodegradability, outstanding biocompatibility, non-toxicity, and superior physical and chemical characteristics [19]. Chitosan and its derivatives demonstrate antibacterial activity against fungi, gram-positive bacteria, and gram-negative bacteria [20]. In recent years, several reviews have explored its antibacterial effects [21]. The antibacterial properties of chitosan have been assessed by measuring the mortality rates of *Escherichia coli* and *Staphylococcus aureus*, focusing on cell wall damage or loss, as well as enzyme and nucleotide leakage from different cellular compartments [22]. Studies have shown that chitosan interacts with both the cell wall and the cell membrane, though not simultaneously, suggesting a specific mechanism in the inactivation of *E. coli* by chitosan [23].

Two formyl groups (-CHO) are present in the chemical molecule glyoxal [24]. Cross-hyperlinking polysaccharides are its typical application [25]. It is cross-linked either via Schiff's base formation between the -NH_2_ group of chitosan and the -CHO of glyoxal or by acetal formation between the -CHO of glyoxal and the -OH of chitosan [26]. Additionally, it was observed that the combination of chitosan and tannic acid, when cross-linked using glyoxal and lyophilized, results in the formation of porous biocompatible structures [27].

Anthracene succinimide derivatives are a class of compounds that can be synthesized by reacting anthracene with succinimide [28, 29]. These derivatives have various applications in organic chemistry, materials science, and pharmaceuticals due to their unique structural properties. One common use of these derivatives is in the development of organic semiconductors for electronic devices [30].

A naturally occurring cyclic oligosaccharide, betacyclodextrin (β-CD) is composed of seven glucose units connected by glycosidic linkages. It is part of the cyclodextrin family, which also includes α-CD (six glucose units) and γ-CD (eight glucose units). The unique structure of β-CD gives it several interesting chemical and physical properties, making it useful in various applications [31].

Because of their small size, metal oxide nanoparticles (MO NPs) have a high surface area-to-volume ratio. This property makes them ideal for catalytic applications and adsorption of pollutants [32]. MO NPs exhibit unique optical properties, such as plasmonic effects and quantum confinement effects, which make them suitable for various applications in optics, sensors, and photonics [33]. Some MO NPs such as silver, zinc oxide titanium oxide, and alumina oxide NPs, have been shown to exhibit antibacterial properties, making them useful in medical applications and antimicrobial coatings [34]. All things considered, MO NPs provide a flexible platform with a variety of characteristics that may be customized for particular uses in environmental remediation, electronics, catalysis, and medicine [35, 36].

Chitosan, a biopolymer derived from chitin, has attracted considerable interest in the field of antimicrobial materials due to its natural origin, biodegradability, non-toxicity, and broad-spectrum antimicrobial properties [37]. However, its antimicrobial activity is often limited by its solubility and stability under physiological conditions. To overcome these limitations, chitosan can be chemically modified to enhance its properties, including antimicrobial efficacy. Chitosan derivatives, such as those modified with glyoxal and AS, offer promising solutions for improving the antimicrobial performance of chitosan-based materials. Glyoxal, a reactive dialdehyde, is commonly used to crosslink chitosan, leading to increased structural stability and improved resistance to microbial attack [38]. The introduction of the AS moiety further enhances antimicrobial activity due to its aromatic structure, which can interact with microbial cell membranes, causing disruption and ultimately inhibiting bacterial and fungal growth. The combination of these two modifications glyoxal crosslinking and incorporation of AS has shown promise in improving the bactericidal and fungicidal activity of chitosan derivatives. These materials hold promise for use in advanced antimicrobial coatings, drug delivery systems, and wound dressings, where superior antimicrobial efficacy is essential [30].

In this study, Anthracene succinimide derivatives are known for their π-conjugated aromatic systems and have been widely explored in material science for their photophysical and electronic properties. In the present study, the N-amino-9,10-dihydro-anthracene-9,10-α,β-succinamide (AS) moiety was strategically incorporated into the chitosan backbone via glyoxal crosslinking to enhance the structural rigidity, conjugation, and antimicrobial performance of the resulting polymer (Chs-Gly-AS). The electron-rich anthracene ring is expected to interact with microbial cell membranes, contributing to membrane disruption, while the succinimide group enhances hydrogen bonding and interaction with biological targets. Thus, its inclusion not only imparts functional benefits related to bioactivity but also adds potential optical and physicochemical characteristics relevant for biomedical applications such as antimicrobial coatings and drug carriers. Additionally, the resulting polymer was modified with β-CD and nanoparticles such as ZnO, TiO_2_, and Al_2_O_3_ to enhance the characteristics of the original polymer (Chs-Gly-AS). This modification of the Chs-Gly-AS polymer was achieved through a straightforward, cost-effective, and eco-friendly approach to create a material with high crystallinity for various applications. These polymers were used as antimicrobial acativity.

## Materials and methods

### Materials

Chs polymer (with a deacetylation level of at least 90%), Glyoxal, and β-CD polymer were acquired from Merck Company in Germany. Glacial acetic acid (AcOH), dimethylformamide (DMF), and nanoparticles (>97% purity), **Zinc Oxide (ZnO) nanoparticles**: <50 nm, **Titanium Dioxide (TiO₂) nanoparticles (anatase phase)**: <25 nm, **Aluminum Oxide (Al₂O₃) nanoparticles**: ~30–60 nm were provided by Aldrich, Milwaukee, Wisconsin, USA.

#### Synthesis of N-amino-9,10-dihydro-anthracene-9,10-α,β-succinamid (AS) 3

**Scheme 1:**
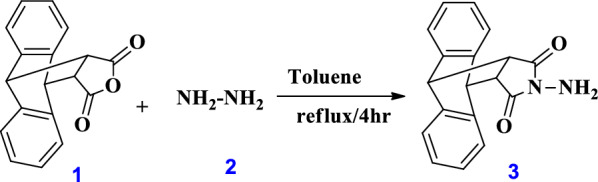
Synthesis of N-amino-9,10-dihydro-anthracene-9,10-α,β-succinamide (AS) via the reaction of 9,10-dihydro-anthracene-9,10-α,β-succinic anhydride with hydrazine hydrate

9,10-dihydro-anthracene-9,10-α,β-succinic anhydride **1** (2.5 g) reacted with hydrazine hydrate **2** (3.2 ml) in boiling toluene for 4 h to form white precipitate. Then, the generated precipitate was crystallized by chloroform to yield AS **3** as only compound in 91 % yield, melting point = 282-284 °C Scheme 1. The ^1^H-NMR (DMSO) *δ* (ppm) = 3.17- 3.20 (d, d, 2H), 4.77 (d, d, 2H), 7.12-7.46 (m, 8H, Ar-H). ^13^C NMR (100 MHz, DMSO-*d*6): *δ* 43.65, 44.83, 44.98, 124.77, 125.24, 127.15, 128.75, 129.51, 129.97, 130.94, 172.60, 174.35.

#### Synthesis of Chitosan- glyoxal- anthracene sucinimide (Chs-Gly-AS) 6

**Scheme 2:**
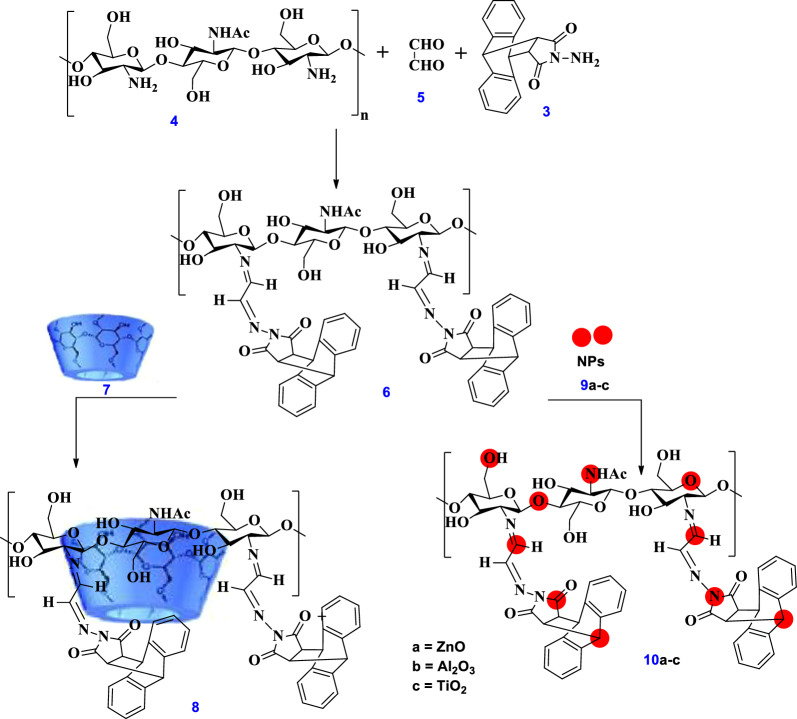
Synthetic route for the preparation of Chs-Gly-AS polymer and its modified derivatives, including Chs-Gly-AS/β-CD and Chs-Gly-AS/nanoparticles (ZnO, Al₂O₃, TiO₂)

Chs **4** (0.5 g) was dissolved in 50 ml of AcOH (1% v/v) for 20 mins at room temperature. At the same time, AS **3** (0.5 g) was dissolved in 20 ml DMF. Then, add solution of chitosan and AS at once into glyoxal **5** solution (1.0 ml in 20 ml DMF) at room temperature (RT) for 16 h, Scheme 2. The mixture was subjected to filtration, producing a brown solid that was subsequently rinsed with distilled water. After that, the obtained polymer **6** was permitted to air dry at RT.

#### Synthesis of Chitosan- glyoxal- anthracene sucinimide /β-CD (Chs-Gly-AS/β-CD) 8

For two hours at 50 °C, the Chs-Gly-AS polymer **6** was dissolved in 40 ml of DMF until a clear solution was afforded, Scheme 2. Add the 2.0 g β-CD **7** solution in 20 ml DMF to Chs-Gly-AS **6** solution for 20 hours. Washing the reaction mixture by using Dist. water after it had been filtered to produce a pale yellow precipitate. After that, the resultant polymer **8** was left to cure at RT.

#### Synthesis of Chitosan- glyoxal- anthracene sucinimide/Nano particles (Chs-Gly-AS / NPs) 10a-c

The concentration of 3% nanoparticles used in this study is consistent with concentrations commonly employed in nanocomposite research. At this concentration, the observed cytotoxicity is minimal, and it is within an acceptable range compared to studies that have used higher concentrations. This approach ensures the enhancement of the composite properties while maintaining cellular viability [39, 40].

For 40 minutes at RT, 1.0 g of Chs-Gly-AS **6** was agitated in 40 ml of DMF until a clear solution was achieved. Scheme 2 was then used to ZnO, Al_2_O_3_ or TiO_2_ NPs solution (0.03 g) with the Chs-Gly-AS **6** solution in 20 ml DMF for 20 h. Following filtering, the reaction mixture produced a pale yellow, dark brown, and pale brown precipitate, respectively. The precipitates that were produced were then cleaned using purified water. Following creation, the polymers **10a-c** were dried at RT, respectively.

### Charaterization and antimicrobial evaluation

#### ^1^H-NMR spectroscopy

Proton nmr spectra were recorded at 400 MHz using oxford and Varian mercury instruments and DMSO was used as a solvent. The chemical shifts are given on the δ scale and TMS was used as an internal standard. The absorption peaks are indicated by s (singlet), d (doublet), dd(double of doublets), t (triplet), q (quartet) and m (multiple).

#### Fourier-transform infrared spectroscopy (FT-IR)

FT-IR is a method employed to examine the chemical makeup of a sample by assessing the absorption or emission of infrared radiation. This technique yields critical insights into the molecular structure, bonding characteristics, and overall composition of a substance, establishing it as an essential instrument for research and analytical purposes. The analysis was performed using a Jasco Model 4100 infrared spectrometer, produced in Japan. To enable FT-IR spectroscopy focused on exploring the structure of the synthesized derivatives at ambient temperature, samples were prepared on potassium bromide (KBr) disks.

#### X-ray diffraction (XRD)

XRD is an effective method for examining the crystallite size and phase composition of various materials, such as polymers. At 25 °C, XRD patterns were recorded within the 5°–80° range.

#### Transmission electron microscopy (TEM)

TEM is an advanced imaging technique that utilizes a high-energy electron beam transmitted through an ultra-thin specimen to produce high-resolution images at the nanoscale. The interaction between the electrons and the sample generates detailed information about the sample's internal morphology, crystallography, and composition. TEM is particularly effective for observing nanoparticles, polymeric matrices, and nanocomposite structures, providing magnifications up to several million times with nanometer or even sub-nanometer spatial resolution.

#### Antimicrobial study

The antimicrobial activity of Chs-Gly-AS and its derivative polymers were studied by the cup diffusion agar method. The four representative test microbes used were Staphylococcus aureus ATCC 6538-P (G + ve), Escherichia coli ATCC 25933 (G-ve), Candida albicans ATCC 10231(yeast) and Aspergillus niger NRRL-A326 (fungus). Nutrient agar plates were heavily inoculated regularly with 0.1 ml of 105–106 cells/ml in case of bacteria and yeast. Potato dextrose agar plates seeded by 0.1 ml (106 cells/ml) the fungal inoculum was used to evaluate the antifungal activities. 100 µl of samples dissolved in DMSO (20 mg in 2 ml DMSO) were placed in initiated holes in inoculated plates. Then plates were kept at low temperature (4 °C) for 2–4 h to allow maximum diffusion. The plates were then incubated at 37 °C for 24 h for bacteria and at 30 °C for 48 h in upright position to allow maximum growth of the organisms. The antimicrobial activity of the test agent was determined by measuring the diameter of zone of inhibition expressed in millimeter (mm). The experiment was carried out more than once and mean of reading was recorded. The antimicrobial activity of chitosan and its derivative polymers was evaluated using the cup diffusion agar method. Four representative test microorganisms were used: Staphylococcus aureus ATCC 6538-P (Gram-positive bacteria), Escherichia coli ATCC 25933 (Gram-negative bacteria), Candida albicans ATCC 10231 (yeast), and Aspergillus niger NRRL-A326 (fungus). Nutrient agar plates were inoculated with 0.1 ml of bacterial or yeast suspension (10^5–10^6 cells/ml), while potato dextrose agar plates were seeded with 0.1 mL of fungal inoculum (10^6 cells/ml) to assess antifungal activity. Samples (100 µl) dissolved in DMSO (20 mg in 2 ml DMSO) were added to pre-formed wells in the inoculated agar plates. The plates were incubated at 4 °C for 2–4 h to allow diffusion, followed by incubation at 37 °C for 24 h for bacterial growth and at 30 °C for 48 h for fungal growth. The antimicrobial activity was measured by the diameter of the zone of inhibition in millimeters (mm). The experiment was repeated multiple times, and the mean values were recorded.

## Result and discussion

### Preparation of Chs-Gly-AS derivatives

Using ethanedial oxalaldehyde (glyoxal) as a cross linking between chitosan and N-amino-9,10-dihydro-anthracene-9,10-α,β-succinamid afforded 1,2-ethanediimine derivatives such as Chs-Gly-AS. Additionally, using βCD and nanoparticles for improving the physical and chemical properties of the main polymer (Chs-Gly-AS). The generated polymer are consider promising polymer for high biological activities and various applications.

### XRD analysis

**Fig. 1:**
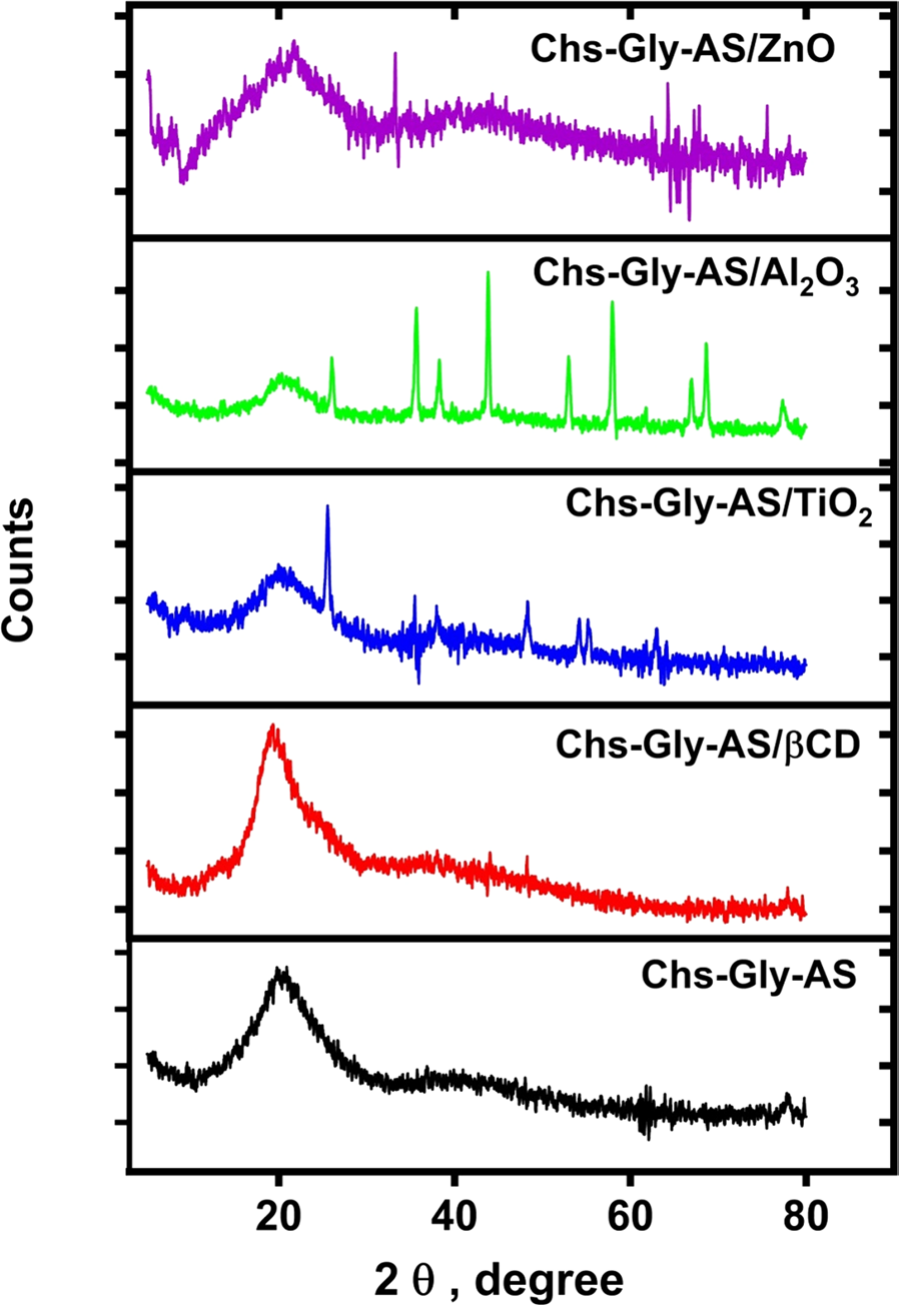
XRD patterns of Chs-Gly-AS polymer, Chs-Gly-AS/β-CD, Chs-Gly-AS/Al₂O₃ NPs composite, Chs-Gly-AS/TiO₂ NPs composite, and Chs-Gly-AS/ZnO NPs composite

X-ray diffraction (XRD) is a powerful analytical technique widely used to investigate the crystallographic structure, crystallite size, and phase composition of various materials, including polymer-based systems. In this study, XRD measurements were conducted at 25  °C across a scanning range of 2θ = 5° to 80° (Fig. 1).

**Chitosan (Chs):** Pure chitosan exhibited two main diffraction peaks at 2θ = 8.6° and 20°, consistent with its semi-crystalline nature, as reported in the literature [12].

**Chs-Gly-AS Polymer (6):** This polymer was synthesized via chemical modification of the amino group in chitosan. The XRD pattern showed a new prominent peak at 18° and a weak peak at 37°, indicating changes in the crystalline structure. The polymer exhibited a crystallinity of 65% and a crystal size of 2.6 nm. The intensity of its peaks was higher than that of the nanoparticle-based composites but lower than the Chs-Gly-AS/β-CD pseudopolyrotaxane.

**Chs-Gly-AS/β-CD Complex (8):** This pseudopolyrotaxane complex demonstrated enhanced crystalline characteristics, as evidenced by a sharp and intense diffraction peak at 22.5°. It had the highest crystallinity (80%) among the tested samples and the largest crystallite size of 3.39 nm, suggesting the inclusion of β-CD significantly improves ordering within the polymer matrix.

**Chs-Gly-AS/Al₂O₃ NPs Composite (10a):** After embedding Al₂O₃ nanoparticles, the XRD profile of composite 10b showed reduced crystallinity (56%) and a smaller crystal size of 2.4 nm. A previously broad peak became narrower with diminished intensity, and distinct sharp peaks appeared at 38°, 39°, 43°, 58°, 69°, 72°, 73°, and 79°, which can be attributed to the crystalline nature of Al₂O₃.

**Chs-Gly-AS/TiO₂ NPs Composite (10b):** The addition of TiO₂ nanoparticles resulted in further reduction in crystallinity (52%) and the smallest crystal size among the composites (1.9 nm). The XRD pattern displayed a broader halo with decreased intensity, along with sharp diffraction peaks at 26°, 38°, 40°, 51°, 58°, 59°, and 62°, corresponding to the TiO₂ phase.

**Chs-Gly-AS/ZnO NPs Composite (10c):** Upon incorporating ZnO nanoparticles, the resulting composite showed a crystallinity of 54% and a crystal size of 2.3 nm. A noticeable broadening and slight shift in the main peak were observed, with increased intensity. Additionally, sharp diffraction peaks emerged at 10°, 27°, 38°, 42°, 63°, and 66°, indicating the presence of ZnO crystalline domains.

### FT-IR analysis

**Fig. 2:**
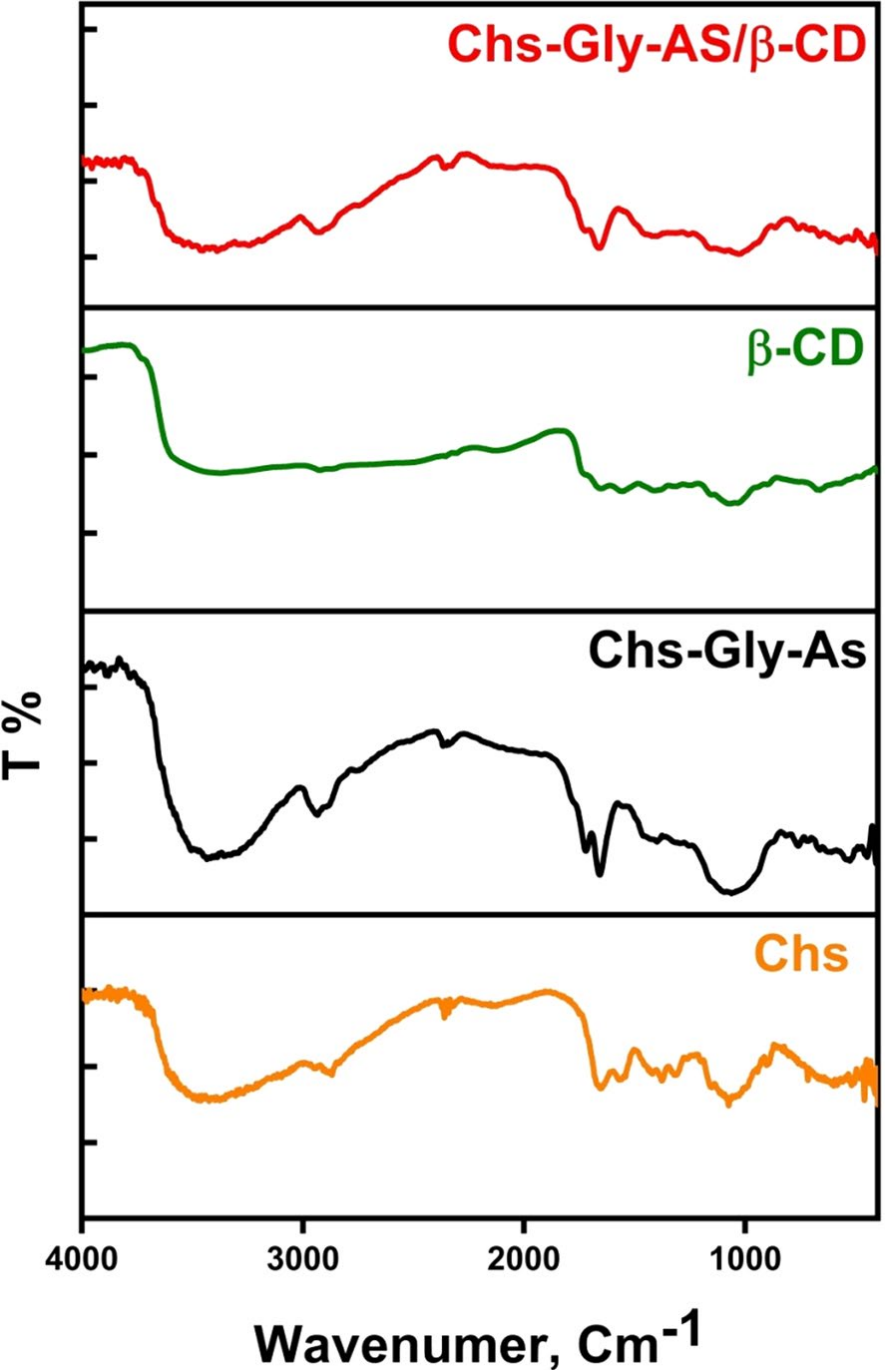
FT-IR of Chs, Chs-Gly-AS polymer, β-CD and Chs-Gly-AS/β-CD

The FT-IR spectrum of the Chs-Gly-AS polymer 6 is depicted in Fig. 2. The C-H hydrocarbon bond was detected at 2910 cm^−1^ in the chitosan spectrum, while the peak for the -OH group was found to overlap with the N–H stretching band at 3330 cm^−1^. Additionally, peaks for the amino group (-NH_2_) and the carbonyl group (-C = O) were recorded at 1600 cm^−1^ and 1659 cm^−1^, respectively [3]. The Chs-Gly-AS polymer spectrum exhibited two overlapping NH-amidic peaks at 3450 cm^−1^, confirming the successful synthesis of the polymer. Moreover, after the cleaning procedure, the spectrum of Chs-Gly-AS polymer 6 did not display the characteristic band associated with glyoxal in the 1200–1600 cm^−1^ region, suggesting that there are no significant residues of free glyoxal present. These observations affirm the successful formation of Chs-Gly-AS 6 through the reaction of chitosan and glyoxal under the defined reaction conditions.

The differences between Chs-Gly-AS and the Chs-Gly-AS/β-CD complex are depicted in Fig. 1, based on FTIR spectral analysis. It was observed that the absorption band of the -OH group is significantly more intense than that of β-CD [41]. Additionally, the symmetric stretching frequency of v[OH] was found to shift to a higher frequency, while the CH-aliphatic peak shifted to a lower frequency when compared to β-CD. The bending vibrations for [CH_2_-O] and [C–O–C] were also noted at lower frequencies of 1320 cm^−1^ and 1080 cm^−1^, respectively. These results confirm that the interaction between β-CD and Chs-Gly-AS 6 leads to the formation of a pseudopolyrotaxane polymer. This can be explained by the fact that the introduction of Chs-Gly-AS 6 into the electron-rich cavity of the cyclodextrin results in an increase in frequency [12]. Table 1 provides a summary of the absorbance band differences among Chs-Gly-AS, β-CD, and the Chs-Gly-AS/β-CD polymer.

**Table 1:** FT-IR changes of β-CD, 6 polymer, and 8 polymer

Functional groups	Wave number, cm^1^				
	∆ν_1_	Chs-Gly-AS	Chs-Gly-AS/β-CD	β-CD	∆ν_2_
ν[OH,NH] symmetric	+ 70	3330	3400	3389	− 11
ν [CH aliphatic]	-95	2910	2815	2925	− 110
ν [C-O]	+ 9	1659	1668	–	–
ν [CH_2_-OH]	+ 100	1220	1320	1159	+ 161
ν [C–O–C]	-30	1110	1080	1028	+ 52
ν [C-N]	+ 100	3450	3550	–	–

∆ν_1_ = ν(Chs-Gly-AS/β-CD) –ν(Chs-Gly-AS), ∆ν_2_ = ν(Chs-Gly-AS/βCD) –ν( β-CD)

Figure 2 was showed the difference between Chs-Gly-AS and Chs-Gly-AS/β-CD via FTIR pattern. We discovered that the -OH group’s absorption band is higher and more intense than the βCD’s [41, 42]. Additionally, the v[OH] symmetric stretching migrated to a higher frequency and the CH-aliphatic peak to a lower frequency in comparison to βCD. Additionally, the bending vibrations [CH_2_-O] and [C–O–C] were displaced at lower frequencies of 1320 and 1080 cm^−1^, respectively. These findings confirmed that the interaction between βCD and Chs-Gly-AS **6** polymer produced pseudopolyrotaxane polymer **8**. The following explanation applies to this finding: When the Chs-Gly-AS **6** is introduced into the CDs electron-rich cavity, the frequencies increase [12]. Table 1 summarizes the differences in absorbance bands of Chs-Gly-AS, βCD and Chs-Gly-AS/βCD polymer.

**Fig. 3:**
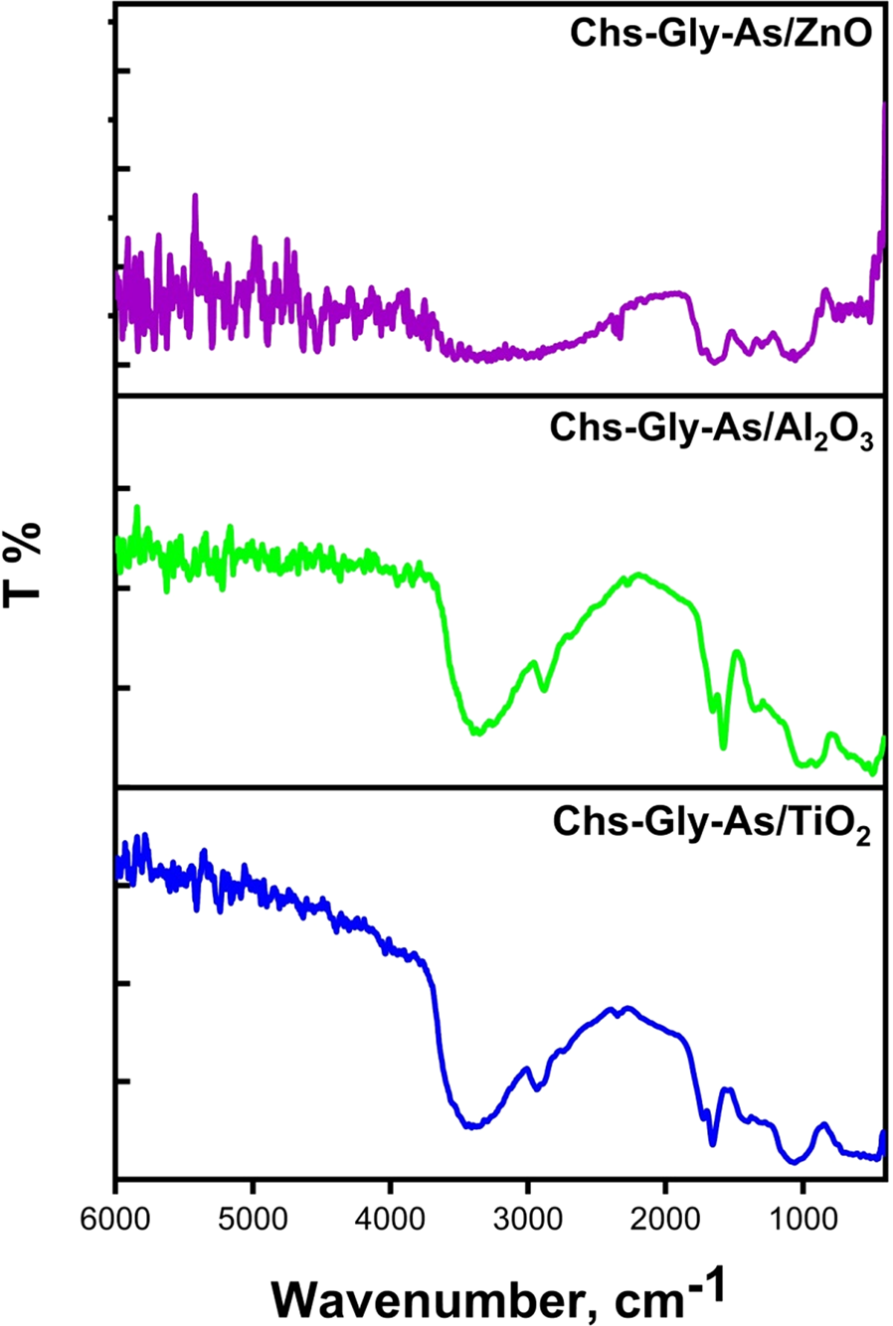
FT-IR of Chs-Gly-AS/Al₂O₃ NPs composite, Chs-Gly-AS/TiO₂ NPs composite, and Chs-Gly-AS/ZnO NPs composite

The FT-IR spectra of the Chs-Gly-AS/ZnO NPs 10a polymer, as illustrated in Fig. 3, reveal a significant absorption band corresponding to the stretching vibrations of hydroxyl groups at 3500 cm^−1^, characterized by an expanded peak. Additionally, the absorption band observed at 2780 cm^−1^ is attributed to the symmetric stretching of the aliphatic C-H bonds present in the Chs component of the polymer mixture. Following the incorporation of ZnO NPs, this absorption band notably shifts and intensifies to 2800 cm^−1^. The absorption band at 1625 cm^−1^ is associated with the C = O stretching vibration. Furthermore, the absorption bands at 1315 cm^-1^ and 1040 cm^−1^ correspond to the stretching vibration of the (CH_2_-OH) group and the bending vibration of the (C–O–C) group, respectively. The findings presented in Fig. 3 provide compelling evidence for the successful doping of the polymer blend with ZnO NPs. A summary of the variations in absorption bands among the Chs-Gly-AS polymer, the Chs-Gly-AS/ZnO NPs composite, and the ZnO NPs is provided in Table 2.

**Table 2:** FT-IR changes of 6 polymer, 10a composite and ZnO NPs

Functional groups	Wave number, cm^1^				
	∆ν_1_	Chs-Gly-AS	Chs-Gly-AS/ZnO NPs	ZnO NPs	∆ν_2_
ν[OH,NH] symmetric	+ 170	3330	3500	3333	+ 167
ν [CH aliphatic]	− 130	2910	2780	–	–
ν [C-O]	− 34	1659	1625	–	–
ν [CH_2_-OH]	+ 95	1220	1315	1393	− 78
ν [C–O–C]	− 70	1110	1040	1090	− 50
ν [C-N]	− 40	3450	3410	–	−

**∆ν**_1_ = ν(Chs-Gly-AS/ZnO NPs) –ν(Chs-Gly-AS), **∆ν**_2_ = ν(Chs-Gly-AS/ZnO NPs)- ν(ZnO NPs)

The FT-IR spectra presented in Fig. 3 for the Chs-Gly-AS/Al_2_O_3_ NPs 10b polymer demonstrate a significant absorption band corresponding to hydroxyl group stretching vibrations at 3420 cm^−1^, characterized by an expanded peak. Additionally, the symmetric stretching of the aliphatic C-H bonds in the Chs polymer mixture results in an absorption band at 2670 cm^−1^. Following the doping with Al_2_O_3_ NPs, this absorption band experiences a notable shift and intensification to 2790 cm^−1^ [43]. Furthermore, the absorption band at 1650 cm^−1^ is attributed to C = O stretching vibrations. The bands observed at 1380 cm^−1^ and 1000 cm^−1^ indicate the bending of the (C–O–C) group and the stretching vibration of the (CH_2_-OH) group, respectively. These results strongly support the incorporation of the polymer blend into the Al_2_O_3_ NPs, as depicted in Fig. 3. A summary of the variations in absorption bands among the Chs-Gly-AS polymer, the Chs-Gly-AS/Al_2_O_3_ NPs composite, and the Al_2_O_3_ NPs is provided in Table 3.

**Table 3:** FT-IR changes of 6 polymer, 10b composite and Al_2_O_3_ NPs

Functional groups	Wave number, cm^1^				
	∆ν_1_	Chs-Gly-AS	Chs-Gly-AS/Al_2_O_3_	Al_2_O_3_ NPs	∆ν_2_
ν[OH,NH] symmetric	+ 90	3330	3420	3600	+ 167
ν [CH aliphatic]	− 240	2910	2670	–	–
ν [C-O]	− 9	1659	1650	1700	− 50
ν [CH_2_-OH]	− 220	1220	1000	–	–
ν [C–O–C]	+ 270	1110	1380	–	–
ν [C-N]	− 50	3450	3400	–	–
ν [Al–O–Al]	–	–	–	600	–

**∆ν**_1_ = ν(Chs-Gly-AS/Al_2_O_3_) –ν(Chs-Gly-AS), **∆ν**_2_ = ν(Chs-Gly-AS/Al_2_O_3_)- ν(Al_2_O_3_ NPs)

The FT-IR spectra presented in Fig. 3 for the Chs-Gly-AS/TiO_2_ 10c NPs polymer exhibit a significant absorption band corresponding to hydroxyl group stretching vibrations at 3470 cm^−1^, characterized by an expanded peak. The symmetric stretching of the aliphatic C-H bonds in the Chs component results in an absorption band at 2780 cm^−1^. Following the incorporation of TiO_2_ NPs, this absorption band experiences a notable shift and intensification to 2600 cm^−1^. Additionally, the absorption band at 1700 cm^−1^ is attributed to C = O stretching vibrations. The bands observed at 1420 cm^−1^ and 1070 cm^−1^indicate the bending of the (C–O–C) group and the stretching vibration of the (CH_2_-OH) group, respectively. These findings strongly support the incorporation of the polymer blend into TiO_2_ NPs, as the FTIR analysis did not detect the Ti–O, Ti–OH, and -OH peaks at 440, 1610, and 3390 cm^−1^ in the TiO_2_ NPs [44]. A summary of the differences in absorption bands among the Chs-Gly-AS polymer, the Chs-Gly-AS/TiO_2_ NPs composite, and the TiO_2_ NPs is provided in Table 4.

**Table 4:** FT-IR changes of 6, 10c composite and TiO_2_ NPs

Functional groups	Wave number, cm^1^				
	∆ν_1_	Chs-Gly-AS	Chs-Gly-AS/TiO_2_	TiO_2_ NPs	∆ν_2_
ν[OH,NH] symmetric	+ 140	3330	3470	–	–
ν [CH aliphatic]	− 130	2910	2780	–	–
ν [C-O]	+ 41	1659	1700	–	–
ν [CH_2_-OH]	− 150	1220	1070	–	–
ν [C–O–C]	+ 310	1110	1420	–	–
ν [C-N]	− 20	3450	3430	–	–
ν [O-Ti–O]	–	–	–	1100	–
ν [C-O-Ti]	–	–	670	–	–

**∆ν**_1_ = ν(Chs-Gly-AS/ TiO_2_) –ν(Chs-Gly-AS), **∆ν**_2_ = ν(Chs-Gly-AS/ TiO_2_)- ν(TiO_2_ NPs)

### Transmission electron microscopy (TEM)

**Fig. 4:**
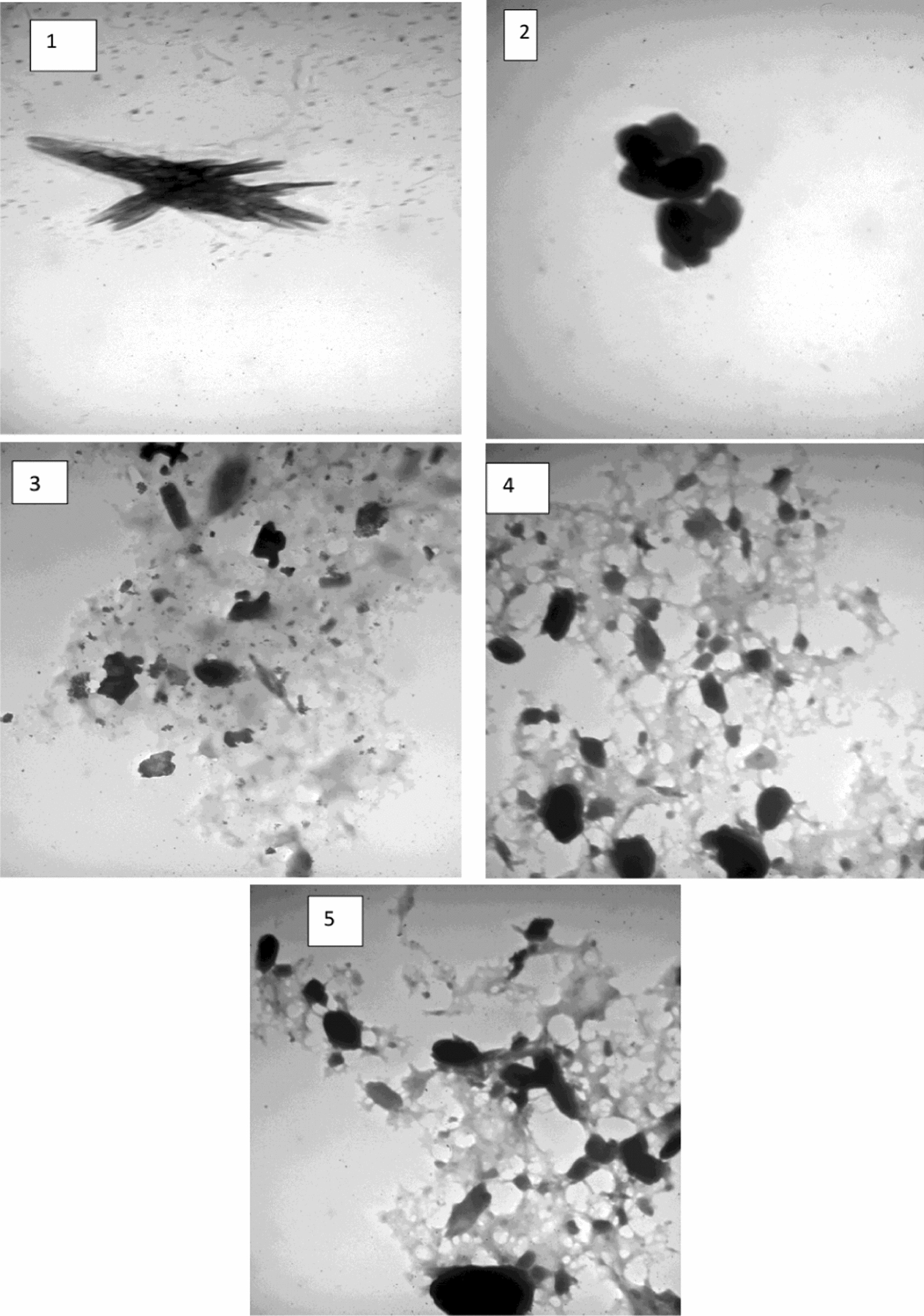
TEM of 1)Chs-Gly-AS, 2)Chs-Gly-AS/β-CD, 3)Chs-Gly-AS/Al₂O₃ NPs composite, 4) Chs-Gly-AS/TiO₂ NPs composite, 5)Chs-Gly-AS/ZnO NPs composite

The TEM image of the Chs-Gly-AS polymer exhibited a relatively uniform and smooth morphology without the presence of dark, electron-dense nanoparticles. The polymer network appeared homogeneous and continuous, indicating the successful formation of Chs-Gly-AS polymer without any detectable phase separation or aggregation. Figure 4.

Incorporation of β-CD into the Chs-Gly-AS resulted in a denser polymer network, as observed by a slight increase in local electron density areas. However, no distinct nanoparticles were identified, suggesting that β-CD was well-integrated into the polymeric matrix at the molecular level, potentially enhancing the crosslinking density and compactness without forming separate nanostructures.

TEM analysis of the Chs-Gly-AS/Al_2_O_3_ composite revealed the presence of uniformly distributed spherical Al_2_O_3_ nanoparticles embedded within the polymeric matrix. The particle size ranged below 50 nm, and slight agglomeration was occasionally observed. Nonetheless, the Al_2_O_3_ NPs were well-encapsulated by the polymer, suggesting good interfacial compatibility between the nanoparticles and the Chs-Gly-AS matrix, which can improve mechanical reinforcement and thermal stability.

The TEM images of the Chs-Gly-AS/TiO_2_ composite displayed well-dispersed, nearly spherical TiO_2_ nanoparticles with diameters ranging from approximately 10 to 30 nm. The dispersion quality was superior compared to the Al_2_O_3_ composite, with minimal nanoparticle aggregation. This excellent dispersion indicates strong interactions between TiO_2_ nanoparticles and the polymeric chains, which may contribute to enhanced photocatalytic and antimicrobial properties.

TEM examination of the Chs-Gly-AS/ZnO composite showed the presence of ZnO nanoparticles with predominantly spherical shapes and some rod-like nanostructures. The particle size was typically between 20 and 50 nm. Although a slight tendency toward nanoparticle aggregation was observed, the ZnO particles were generally well-dispersed within the Chs-Gly-AS polymer. This incorporation suggests potential improvements in UV-blocking, antimicrobial activity, and mechanical performance.

The TEM analysis confirmed the successful formation of the Chs-Gly-AS polymeric matrix and its effective integration with various nanomaterials. While the pristine Chs-Gly-AS and Chs-Gly-AS/β-CD samples exhibited homogeneous, nanoparticle-free morphologies, the incorporation of Al_2_O_3_, TiO_2_, and ZnO nanoparticles resulted in the formation of well-dispersed nanocomposite structures. Among the studied composites, Chs-Gly-AS/TiO_2_ demonstrated the most uniform nanoparticle dispersion with minimal aggregation. Minor nanoparticle clustering was observed in the Chs-Gly-AS/ Al_2_O_3_ and Chs-Gly-AS/ZnO composites but remained within acceptable limits. Overall, the TEM observations validate the successful embedding of nanoparticles into the polymeric matrix, which is expected to significantly enhance the functional properties of the composite.

### Antimicrobial activity

**Fig. 5:**
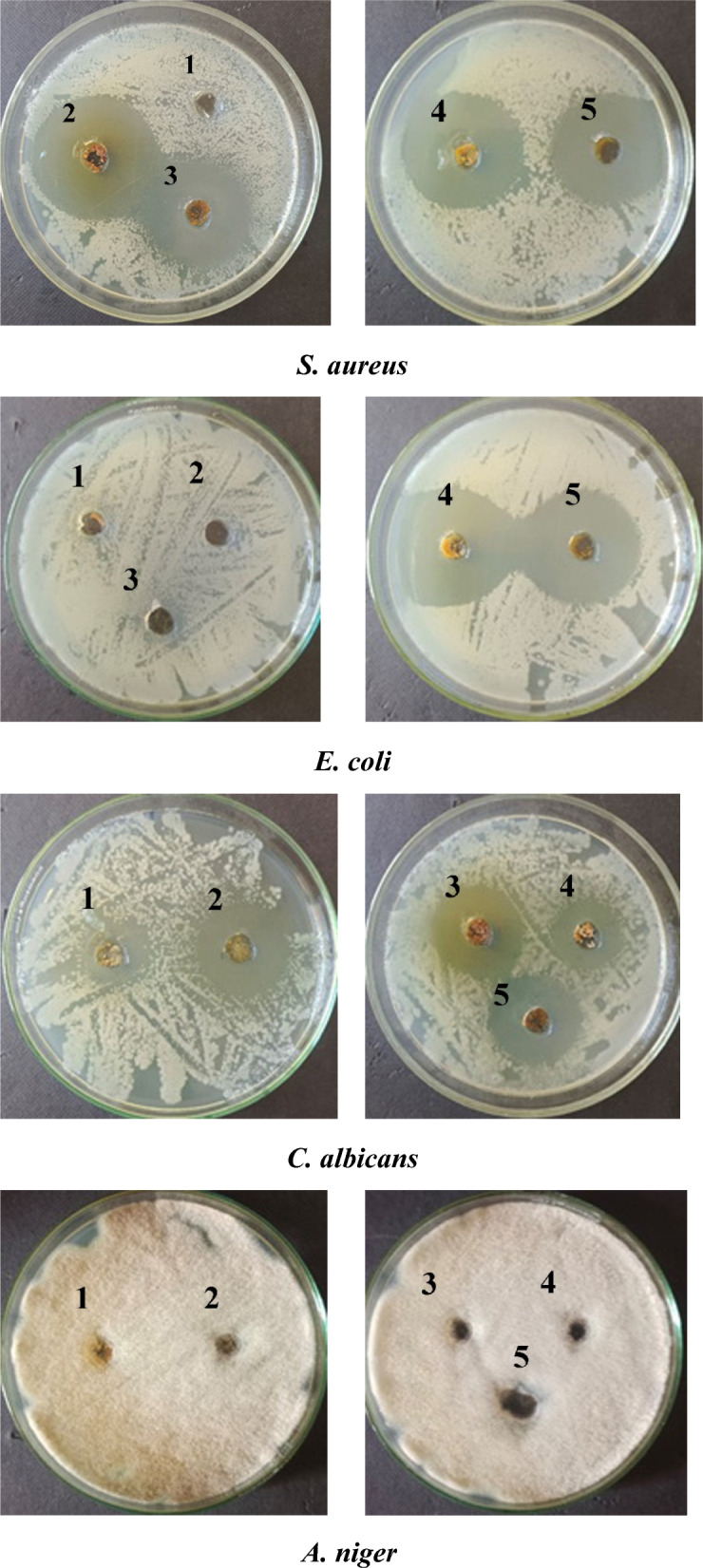
The antimicrobial activity of chitosan-derivative polymers samples studied by the cup diffusion agar method

The results obtained are presented in Fig. 5 and Table 5, which illustrate the antimicrobial properties of chitosan-derivative polymers both prior to and following their modification with nanoparticles. The antifungal and antimicrobial activities of Chs-Gly-AS polymers, modified with nanoparticles such as ZnO, TiO_2_, and Al_2_O_3_, can be attributed to various mechanisms. Notably, TiO_2_ is recognized for its antimicrobial capabilities, primarily through the generation of reactive oxygen species (ROS) including hydroxyl radicals (*OH), superoxide anions (O_2_-), and hydrogen peroxide (H_2_O_2_). These ROS have the potential to compromise bacterial cell membranes, proteins, and DNA, ultimately resulting in the death of bacterial and fungal cells. Research indicates that TiO_2_ nanoparticles can significantly inhibit the growth of S. aureus by disrupting the cell wall, modifying the membrane potential, and causing the leakage of intracellular materials. Furthermore, polymers modified with TiO_2_ nanoparticles have demonstrated inhibitory effects on C. albicans, curtailing its growth and biofilm formation, while the interaction between TiO_2_ and C. albicans leads to increased cellular damage and diminished viability [45]. The rough surface of Al_2_O_3_ NPs in polymer modified Al_2_O_3_ can interact with bacterial and fungal cell membranes cause mechanical damage to the bacterial cell membrane, leading to leakage of cellular contents and ultimately bacterial death. Although, under certain conditions, aluminum ions can be released, which can have a toxic effect on bacteria [46]. These ions can interfere with bacterial enzymatic processes and disrupt the integrity of the cell wall [47]. ZnO NPs has been widely studied for its antimicrobial effects against various pathogens, including Staphylococcus aureus, Escherichia coli, and Candida albicans. The antimicrobial properties of ZnO NPs modified chitosan polymers are attributed to its unique physicochemical properties, such as its small particle size, high surface area, and ability to generate reactive oxygen species (ROS). These ROS can damage bacterial cell membranes, proteins, lipids, and DNA, leading to cell death. ZnO NPs can interact with the bacterial cell membrane, causing alterations in its permeability, leading to leakage of cellular contents and eventual death. ZnO NPs can bind to the polysaccharides in the fungal cell wall, disrupting its structure and function. ZnO NPs may interfere with bacterial metabolism by inhibiting protein synthesis. The positive charge of ZnO NPs can attract negatively charged components on the bacterial cell wall, leading to an accumulation of ZnO NPs on the bacterial surface, which disrupts cellular processes [10]. It has been observed that the Chs-Gly-AS/β-CD derivative exhibits antibacterial activity against microorganisms. This effect may be attributed to the increased amount of free amino acids, which leads to a higher concentration of positive charges. The Chs-Gly-AS/β-CD polymer demonstrates greater antimicrobial activity compared to Chs-Gly-AS, likely due to its enhanced water solubility after complexation with β-CD. The increased solubility may improve contact between the polymer and pathogens, thereby enhancing the antimicrobial effectiveness of the Chs-Gly-ASc polymer [10]. Finally, The observed ineffectiveness (Chs-Gly-As) derivative against fungi could be attributed to several factors such as nanoparticle aggregation that can diminish their surface area, reducing interaction with fungal cells and subsequent reactive oxygen species (ROS) generation [48]. On the other hand, certain fungi possess robust defense mechanisms against oxidative stress, including efficient antioxidant systems that neutralize ROS. If the ROS generated by the nanoparticle components of the Chs-Gly-As complex are insufficient to overwhelm these defenses, the antifungal activity may be compromised.​ Also, the antifungal activity of nanoparticle-based composites is dose-dependent. Insufficient concentrations or inadequate exposure times can lead to suboptimal antifungal effects. Then, if the Chs-Gly-As complex is applied below effective concentrations or for insufficient durations, its antifungal efficacy may be limited [49, 50].

**Table 5:** The antimicrobial actions of chitosan-derivative polymers before and after modification of with nanoparticles

No	Sample name	Clear zone (ϕmm)			
		*Staphylococcus aureus*	*Escherichia coli*	*Candida albicans*	*Aspergillus niger*
1	Chs-Gly-ASc	0	0	29	0
2	Chs-Gly-ASc/TiO_2_	42	0	42	0
3	Chs-Gly-ASc/Al_2_O_3_	41	0	40	0
4	Chs-Gly-ASc/β-CD	41	43	35	0
5	Chs-Gly-ASc/ZnO	44	45	39	0

Chs-Gly-AS/ZnO composites have been developed into hydrogels exhibiting strong antibacterial activity and good biocompatibility, making them suitable for applications like wound dressings and periodontal treatments [51] Chs-Gly-AS/TiO_2_ composites have shown effective antimicrobial activity against pathogens such as *E. coli* and *S. aureus*. These films also improved mechanical properties and reduced light transmittance, making them promising for food packaging applications [52]. The strong evidence which reflects the antimicrobial potential of Chs-Gly-AS/Al₂O₃ composites make them suitable for developing antimicrobial coatings to prevent infections in Medical Devices, ​creating materials that promote healing while preventing microbial growth in Wound Dressings and designing filters that eliminate bacterial contaminants in water Treatment [53, 54]​.

## Conclusion

This study successfully developed novel chitosan-based polymers by utilizing glyoxal as a crosslinking agent between chitosan and anthracene succinimide to form Chs-Gly-AS. The resulting polymer was further modified with β-CD and nanoparticles to generate Chs-Gly-ASc/ β-CD and Chs-Gly-ASc/NPs, respectively. Characterization of these materials using XRD revealed significant changes in crystallinity, confirming the successful incorporation of β-CD and NPs into the Chs-Gly-ASc polymer. FTIR analysis identified the presence of key functional groups, verifying the chemical modifications and crosslinking interactions within the system. The modification of Chs-Gly-ASc polymers with nanoparticles like TiO₂, Al₂O₃, and ZnO significantly enhances their antimicrobial and antifungal activities. This improvement is primarily due to the generation of reactive oxygen species, mechanical disruption of cell membranes, and interference with cellular processes. Additionally, the Chs-Gly-AS/β-CD derivative exhibits superior antimicrobial effects, likely due to increased solubility and higher positive charge density, leading to stronger interactions with pathogens.

## Data Availability

The datasets used and/or analysed during the current study are available from the corresponding author on reasonable request.

## Abbreviations

Chs: Chitosan
MO NPs: Metal oxide nanoparticles
AS: N-amino-9,10-dihydro-anthracene-9,10-α,β-succinamide
XRD: X-ray diffraction
IR: Infrared spectroscopy
Gly: Glyoxal
β-CD: Beta-cyclodextrine
CDs: Cyclodextrine rings
AcOH: Glacial acetic acid
DMF: Dimethylformamide
mm: Millimeters
RT: Room temperature
ROS: Reactive oxygen species
*OH: Hydroxyl radicals
O_2_-: Superoxide anions
H_2_O_2_: Hydrogen peroxide
